# Integrating Care for Mother–Infant Dyads After Preterm Birth: A Qualitative Study of Clinician Perspectives on Feasibility

**DOI:** 10.1089/whr.2023.0098

**Published:** 2023-12-18

**Authors:** Emily F. Gregory, Peter F. Cronholm, Lisa D. Levine, Rinad S. Beidas, Mario P. DeMarco, Ann L. O'Sullivan, Scott A. Lorch, Adya I. Maddox, Katherine Wu, Alexander G. Fiks

**Affiliations:** ^1^ Department of Pediatrics, University of Pennsylvania Perelman School of Medicine, Philadelphia, Pennsylvania, USA.; ^2^ Clinical Futures, Children's Hospital of Philadelphia, Philadelphia, Pennsylvania, USA.; ^3^ PolicyLab, Children's Hospital of Philadelphia, Philadelphia, Pennsylvania, USA.; ^4^ Department of Family Medicine and Community Health, University of Pennsylvania Perelman School of Medicine, Philadelphia, Pennsylvania, USA.; ^5^ Maternal Fetal Medicine Research Program, Department of Obstetrics and Gynecology, University of Pennsylvania Perelman School of Medicine, Philadelphia, Pennsylvania, USA.; ^6^ Department of Medical Social Sciences, Feinberg School of Medicine, Northwestern University, Chicago, Illinois, USA.; ^7^ Department of Family and Community Health, University of Pennsylvania School of Nursing, Philadelphia, Pennsylvania, PA.

**Keywords:** implementation, infant health, interconception care, maternal health, preventive services, postpartum care

## Abstract

**Objective:**

There are gaps in receipt of maternal preventive services in the interconception period. Yet mother–infant dyads have frequent health care visits. Health systems have opportunities to better capitalize on existing visits to address dyad needs, but this possibility has not been fully explored.

**Methods:**

In this qualitative study we conducted semistructured interviews with clinical team members involved with birthing parents or infants after preterm birth. We conducted snowball sampling from teams in pediatrics, obstetrics, and family medicine at two geographically adjacent health systems. Interviews explored perspectives on existing barriers and facilitators to integrating dyad care across adult and infant teams. Interviews were audio-recorded, professionally transcribed, and coded using an integrated approach.

**Results:**

We interviewed 24 physicians, nurses, midwives, and social workers (March–November 2021). Participants identified barriers to integrated care including infrequent communication between clinical teams, which was generalizable to care of the birthing parent or infant as individuals, and additional barriers related to privacy, credentialing, and visit design that were specific to dyad care. To improve integration of dyad care, clinicians proposed adapting a variety tools and procedures currently used in their practices, including electronic health record tools for communication, dedicated roles to support communication or navigation, centralized information on resources for dyad care, referral protocols, identifying dyads for proactive outreach, and opportunities for clinicians to connect face-to-face about shared patients or families.

**Conclusions:**

Clinicians believe existing health care structures and processes can be adapted to address current substantial barriers to integrated dyad care.

## Introduction

The United States is experiencing increased maternal morbidity and mortality, along with substantial racial inequities in birth outcomes.^1,2^ Sixty percent of births are repeat births, highlighting the importance of health promotion during the interconception period, which lasts from birth to the start of a subsequent pregnancy.^3^ Pediatric clinicians see birthing people frequently during this period.^4^

At the level of the mother–infant dyad, there are a median of nine visits for Medicaid-insured dyads in the year after birth, two thirds of which occur in pediatric settings.^4^ Dyad health care during this year is characterized by gaps in recommended maternal care, including low use of universally recommended postpartum care and of follow-up for pregnancy complications.^4–7^ The frequency of pediatric contact in this year coupled with the importance of maternal health in facilitating child health has led to increased pediatric attention to maternal health.

For example, professional guidelines and Medicaid payment both support postpartum depression screening in pediatric settings.^8,9^ A more comprehensive approach to addressing maternal health through infant visits was developed in a family medicine practice collaborative that implemented screening of birthing parents for interconception health needs at all well visits from birth to 2 years.^10^ Family medicine is well suited to address dyad needs, as it can provide both adult and pediatric primary care as well as obstetrical care for low-risk pregnancies. Yet health systems may not be able to rely on family medicine to ensure integrated care for all new families, as 80% of infants in the United States receive primary care from pediatricians, and family medicine prenatal services are declining.^11,12^

In reality, visits for interconception dyads are likely spread across multiple settings, including pediatrics, neonatology, internal medicine, family medicine, and obstetrics.^4^ If priorities and follow-up needs are not communicated across settings, involvement of multiple teams may limit health systems' ability to support maternal and infant health. Although health systems have addressed integration of care for individuals who receive care from multiple specialists, there has been little attention to integrating care for mother–infant dyads.^13,14^ In this qualitative study, we explored clinician perspectives on the feasibility of integrated care for dyads.

## Methods

### Setting, interview guide, and sampling

This qualitative study was conducted at two large urban geographically adjacent academic health systems. One is a pediatric health system and the other is a health system that provides services, including pediatric and adult primary care and obstetrical care.

Interviews were part of a larger project considering adaptation of care management as a dyad care strategy after preterm birth. Care management is defined as a “team-based, patient-centered approach designed to assist patients and their support systems in managing medical conditions more effectively.”^15^ Care management programs vary depending on the needs of different clinical settings, but typically add clinical roles specifically tasked with providing care planning and care navigation.^13^ The current project focused on care management as a potential strategy to improve interconception care because of its evidence base in integration of care for individuals.^13,16^

The interview guide broadly explored experiences of clinical team members caring for women and infants after preterm birth. This article focuses on interview content generated primarily by questions about collaboration across teams caring for postpartum women and infants and about how care management might intersect with existing team structures.

Participants represented a convenience sample recruited from the two health systems. The study team generated a list of clinicians from teams most involved in care of interconception dyads. This list included people from pediatrics, obstetrics, and family medicine. The pediatric sample included representation from primary care, neonatology, and care management. The family medicine sample focused on physicians who included obstetrical care in their practice.

Neither obstetrics nor family medicine had dedicated roles for care management for new parents or infants, but social workers did provide some care navigation in their settings. We added to this list through snowball sampling, in which we asked participants for suggestions of other clinical team members who might have distinct perspectives or experiences with these topics. We focused on clinicians to explore aspects of daily clinical care influencing feasibility.

This study was reviewed by the Institutional Review Board at the Children's Hospital of Philadelphia and was considered exempt.

### Recruitment and interviews

Recruitment occurred by e-mail. All interviews occurred by phone or video, in part because interviews occurred during a period of evolving COVID-related restrictions. Interviews were conducted privately. Participants were offered gift cards to offset the burdens of participating in research.

Interviews were semistructured, following a guide but allowing interviewers to clarify or explore responses. Interviews were conducted by one of two researchers (E.F.G. and K.W.) who had previous qualitative experience in areas relevant to maternal–child health. Interviewers were oriented to the research project and the interview guides. Interviews were assigned so that there was no pre-existing relationships or collaboration between interviewers and participants.

Interviewers introduced themselves by describing their role with this research project. To contextualize the sample, clinicians were asked to report their department, clinical credential, and time in their profession and in current role. Interviews were audio-recorded and transcribed by a professional transcription service, and continued until we reached thematic saturation.

### Analysis

We applied an integrated approach to coding, combining both inductive and deductive coding.^17^ We started with an *a priori* codebook informed by our interview guide (*e.g.,* care navigation, health care access) and allowed themes to emerge from the data.^18,19^ One model used to consider health system innovation is Donabedian's Model of Health Care Quality.^20^ This model focuses on health care structures and processes as drivers of outcomes. As themes emerged relating to how health systems already integrating care across sites, we applied codes related to health care structure and process, consistent with this model.

Coding was completed by interviewers and one additional study team member (A.I.M.) using NVivo software (Version 12.0, 2018). All coders had training in qualitative research and were oriented to the project. All transcripts were coded by two people, who met to review differences in coding. These differences were used to clarify the coding scheme and were resolved through discussion.^21^

During interviewing and coding, the study team held regular debriefing meetings to review impressions and consider coding scheme revisions. Meeting content was summarized and shared with the study team through memos describing changes and clarifications. Meetings and memos were intended to promote reflexivity (*i.e.,* considering the extent to which our own experiences and beliefs influenced coding).^22^

## Results

We interviewed 24 clinicians (Table 1) in family medicine, obstetrics, and pediatrics. Participants included physicians, registered nurses, nurse practitioners, midwives, and social workers. Interviews occurred from March to November 2021 and lasted a mean of 40 minutes (range 27–52). There was convergence among subgroups on findings.

**Table 1.** Department and Credential of Participants

	Credential				
	Registered nurse	Physician	Social worker	Nurse practitioner (including midwife)	Total
Pediatrics	2	7	2	2	13
Obstetrics		3	2	2	7
Family Medicine		4			4
Total	2	14	4	4	24

We present findings from the interviews in two sections: barriers to dyad care and application of existing strategies to improve dyad care.

### Theme 1: Barriers to integrated dyad care

Participants described barriers to integrated care in four main categories: (1) communication and accountability, (2) privacy, (3) credentialing, and (4) visits for individuals (Table 2). Challenges related to communication were not limited to dyad care but rather also existed in care of individuals. For the other three categories, challenges were more dyad-specific.

**Table 2.** Barriers to Integrated Dyad Care

(1) Communication and accountability	I had to contact someone [in another division] and it was like I didn't know how to get, figure out who they were, what I needed. Thankfully, someone knew, whatever. But it took me like three emails to get to the person because there was no way of figuring that out. *Participant 7, SW, Obstetrics*
	I think we probably have a reliance on the fact that somebody else is picking up on that, but we don't really know. So I could imagine a system – I think historically that this probably is not in place because it's pretty onerous. Well, who is going to own that follow-up and do the phone call or email or whatever the system might be? *Participant 13, MD, Obstetrics*
	I don't know really what happens after they leave [the hospital], in all seriousness. So I mean I would think it would be helpful to have someone there to check in on mom… making sure that she got connected to the resources and followed-up on the resources. *Participant 6, SW, Pediatrics*
	We don't really know what happened to the baby. The baby might've died in the interim because of some sort of complication and you're just seeing [the mother for a postpartum visit] and assuming everything is fine and congratulating them. And we've had very bad experiences with patients as a result. *Participant 9, MD, Obstetrics*
(2) Privacy (EHR governance and HIPAA)	It's hard [for one health care worker] to span a couple of health care systems… they're not going to have medical record access at like five different systems… Technically through Care Everywhere, if you put it in a chart, they should see it. But then you're talking about a chart for parent, versus a chart for the child… And we do occasionally [email about the mother's health], I'd say. But that's – it's rare to do that because there's always, yeah, HIPAA stuff. *Participant 19, MD, Pediatrics*
(3) Credentialing (qualifications, training, safety, liability)	If I ask one of our [pediatric] MAs or nurses to take a [maternal] blood pressure, they kinda look at sideways because it's not what we do. And so, sometimes I've just done it myself. But there's definitely ways that could be automated, that could be a team approach, a standard approach for anyone who had a history of preeclampsia… I think people are worried about the liability. *Participant 1, MD, Pediatrics*
	Breastfeeding support is like kind of one of the biggest things that can sort of spiral to like a mental health need because it can escalate it feels like very quickly for a lot of people…. We might not be as comfortable talking about breastfeeding and feeding with parents who have preterm kids, especially in [the NICU] because I feel like we don't – or I don't want to like step on the toes of the feeding plan a lot of times. *Participant 12, NP, Obstetrics*
	The ability for that – particularly nurse care manager – to really be as effective as possible depends greatly on their collaborating provider… If their collaborating provider is the pediatrician and not the physician caring for the mother, I think the effectiveness of that care management role is gonna be very – it's gonna be limited by that… *Participant 14, MD, Pediatrics*
(4) Visit design	We are just not able to pull together because of resources and processes right now and when the mother is there, you know, tagging co-visits, right? So having the baby be seen and the mom being seen at the same time. And kind of same day LARC insertions and things like that. Just because logistically, it gets very challenging to do that… ideally we would love to figure out and really have a strong program with same day services for both. *Participant 5, NP, Pediatrics*

EHR, electronic health record; HIPAA, Health Insurance Portability and Accountably Act; LARC, Long Acting Reversible Contraception; MAs, Medical Assistant; NICU, neonatal intensive care unit.

#### Communication and accountability

Participants described difficulty communicating across health care settings or divisions, including between inpatient and ambulatory settings, and between different ambulatory divisions or institutions (*e.g.,* obstetrics, pediatrics). Participants reported inconsistent access to contacts or counterparts in other divisions. For example, one participant told us:

I had to contact someone [in another division] and it was like I didn't know how to get, figure out who they were, what I needed. Thankfully, someone knew, whatever. But it took me like three emails to get to the person because there was no way of figuring that out. *Participant 7, SW, Obstetrics*

Uncertainty about how other teams functioned led to a lack of accountability for follow-up of recommended services after a patient or dyad physically left one setting. One participant said:

I think we probably have a reliance on the fact that somebody else is picking up on [worsening symptoms of depression], but we don't really know. So I could imagine a system – I think historically that this probably is not in place because it's pretty onerous. Well, who is going to own that follow-up and do the phone call or email or whatever the system might be? *Participant 13, MD, Obstetrics*

Although these barriers were not specific to dyad care, one participant emphasized why communication across teams is needed in the dyad context:

We don't really know what happened to the baby. The baby might've died in the interim because of some sort of complication and you're just seeing [the mother for a postpartum visit] and assuming everything is fine and congratulating them. And we've had very bad experiences with patients as a result. *Participant 9, MD, OB*

### Privacy

This set of challenges described rules that govern access to electronic health records (EHRs) or communication between teams or institutions. Participant typically attributed these rules to Health Insurance Portability and Accountably Act (HIPAA) regulation. It was not always clear whether participants thought HIPAA prohibited certain types of communication, or whether they thought that institutional concerns about HIPAA compliance would, in practice, create a barrier. For example, one participant said:

[One health care worker is] not going to have medical record access at like five different systems… Technically through [a health information exchange], if you put it in a chart [in one system], they should see it [in the other systems]. But then you're talking about a chart for parent, versus a chart for the child… We do occasionally [email about the mother's health], I'd say. But that's – it's rare to do that because there's always, yeah, HIPAA stuff. *Participant 19, MD, Pediatrics*

#### Credentialing

This theme encompassed a set of concerns about clinician credentialing and qualifications. Clinicians reflected on how training and credentialing for most physicians focus on either adult or pediatric populations, leading to related concerns about safety or liability in providing care that crossed over into the other member of the dyad.

If I ask one of our [pediatric] MAs or nurses to take a [maternal] blood pressure, they kinda look at me sideways because it's not what we do. And so, sometimes I've just done it myself. But there's definitely ways that could be automated, that could be a team approach, a standard approach for anyone who had a history of preeclampsia… I think people are worried about the liability. *Participant 1, MD, Pediatrics*

#### Visit design

Participants noted that visits are designed for individual patients. Because of this, even when services are available on-site that are relevant to maternal care, it may not be feasible to provide maternal services in the context of an infant visit. One pediatrician described efforts to address contraceptive needs of younger mothers, saying:

We are just not able to pull together because of resources and processes right now and when the mother is there, you know, tagging co-visits, right? So having the baby be seen and the mom being seen at the same time. And kind of same day LARC insertions and things like that. Just because logistically, it gets very challenging to do that… ideally we would love to figure out and really have a strong program with same day services for both. *Participant 5, NP, Pediatrics*

### Theme 2: Applicability of existing structure and process to facilitate dyad care

Participants suggested existing strategies used to care for individuals to improve interconception dyad care (Table 3). This content primarily emerged from a section of the interview guide in which participants were provided the Agency for Healthcare Research and Quality (AHRQ) definition of care management and asked to reflect on how a care manager could work across pediatric and adult systems. We present these strategies as (1) structures and (2) processes.

**Table 3.** Applicability of Existing Structures and Processes to Support Interconception Dyad Care

(1) Structure	
EHR to support communication across divisions	If the pediatricians have their access to moms' charts and OBs or family medicine or any internal medicine doctor, just to get a sense of where things are at in some of the more critical cases without violating HIPAA, but they're a joint unit in those cases, that would be nice. *Participant 9, MD, Obstetrics*
Roles to support communication across divisions	I think they would need to have a contact person for each office or department that they could reach out to… Perhaps like different things like that at their disposal. *Participant 10, MD, Family Medicine*
	I know a lot of people in the [prenatal clinic]. I'd probably just pick one to reach out to if I was really concerned [about a postpartum parent]. But as a regular point of contact, I don't know. Not that I know of… I always hate to have one person to rely on, but one role that you could reach out to who's staffed regularly. *Participant 8, MD, Pediatrics*
Centralized resources for dyads	Whether the provider for the child or the provider for the mother identifies a concern, ideally there are resources or a centralized place that they can lean on to help… I think having some sort of process by which any of us could link into resources when a concern is identified certainly would make sense. *Participant 11, MD, Family Medicine*
(2) Process	
Identifying dyads in need of proactive outreach	I just wanna reiterate the [RSV vaccination] model where the patient list is generated, the nurses are doing the outreach. It's happening outside of the patient visit… [because getting important services] shouldn't be just how much time your provider has… So, the more it's automated, I think, the better. *Participant 1, MD, Pediatrics*
	Every kind of like month or so, we are looking at all of our prenatal patients and making sure that we followed up on like their anatomy scan or their third trimester screening or like whatever shenanigans they need done. And then that would also flag if somebody has kind of been missing from care. But we don't have something like that in place for postpartum, but we probably should. *Participant 10, MD, Family Medicine*
Referral protocols	I guess there's a difference to me of being a screening place, a place of screening versus a place of management… I don't know that I would that a pediatrician could manage hypertension in a newly postpartum patient, but I definitely think that's there's processes that could be put into place to safeguard – to have that be the place of detecting it and having it be able to be escalated to the right place to manage it. *Participant 13, MD, Obstetrics*
	You have to be able to assess whether or not the family unit's in crisis and the parent is or the caregiver is unwell and to step in and facilitate the connection between the caregiver and a health system. But getting involved beyond just making the connection or making a suggestion or providing, you know, that's where I think gets blurry. *Participant 15, NP, Pediatrics*
Opportunities for team members to connect face-to-face: Collocation, meetings, and warm hand-offs	We have so many external resources that are truly wonderful, but our collaboration with them is not as good as when it's someone who's in our office… Learning each other's styles and learning how everyone works and operates. *Participant 20, MD, Pediatrics*
	Collaborating with the psychiatrist, but also physically co-locating in the space so that they feel like they're a part of a team together, even if they're providing two different services. So I think co-location to some extent or some sort of consistent person that you know you can either reach out to and they know they can reach out to you and you'll know who they are and having that core true relationship with a person, I think would help improve communication a lot. *Participant 24, MD, Family Medicine*
	When we're getting closer to discharge there could be like a little bit of a warm handoff… “is it okay if we talk about what we've been working on here so they can help follow up with that?” …Obviously with mom's consent… Yes, it will be more work, so it will be another meeting. But… it could just be like a very quick like ten-minute blah, blah, blah. But I think it would be worth it. *Participant 6, SW, Pediatrics*
	I think just making sure that things are being communicated in either team meetings or some kind of huddling or something, so both teams are up to date on what's happening and that there isn't kind of, once again, siloed of, like here's the mom's issues and here's the baby's issues. I think they go hand in hand. So I think however best to huddle together and share that information between both teams is really important. *Participant 5, NP, Pediatrics*
	One of the NICU providers comes to our maternal fetal medicine division meeting once a month to provide follow-up for some of the babies… whoever we request to get a follow-up on. We update the [maternal] chart that way. *Participant 9, MD, OB*

OB, Obstetrician; RSV, Long Acting Reversible Contraception.

#### Structures

Participants suggested EHR-enabled tools for communication, specific roles on the clinical team focused on dyad care, and a centralized directory of resources as structures that could improve integrated dyad care. Notably, although EHR governance was noted as a barrier above, participants also viewed EHR technology as a potential facilitator, as in the following quote:

If the pediatricians have their access to moms' charts and OBs or family medicine or any internal medicine doctor, just to get a sense of where things are at in some of the more critical cases without violating HIPAA, but they're a joint unit in those cases, that would be nice. *Participant 9, MD, Obstetrics*

One participant advocated for developing formal roles to support inter-team collaboration by saying:

I know a lot of people in the [prenatal clinic]. I'd probably just pick one to reach out to if I was really concerned [about a postpartum parent]. But as a regular point of contact, I don't know. Not that I know of… I always hate to have one person to rely on, but one role that you could reach out to who's staffed regularly. *Participant 8, MD, Pediatrics*

The need for dedicated roles to support communication across settings was also alluded to aforementioned when a participant noted that coordinating follow-up after hospital discharge was “onerous” and that no one was specifically tasked with this work. Although we asked about care management, participants did not indicate any credential or title was favored for these roles.

Participants also thought that a centralized resource directory for dyads could improve care.

Whether the provider for the child or the provider for the mother identifies a concern, ideally there are resources or a centralized place that they can lean on to help… I think having some sort of process by which any of us could link into resources when a concern is identified certainly would make sense. *Participant 11, MD, FM*

#### Processes

Processes suggested to improve dyad care included proactively developing and managing a list of patients or dyads to ensure receipt of recommended services (empanelment),^23^ establishing criteria for signs or symptoms that warrant consultation with other clinical teams through clear referral protocols, and ensuring opportunities for members of different clinical teams to connect face-to-face through strategies such as co-location of teams or meetings or warm hand-offs in which members of multiple teams have dedicated time, or clear expectations, for discussion of shared patients or families.^24^

Empanelment was described as improving follow-up for patients or families with low engagement, as in the following quote:

Every kind of like month or so, we are looking at all of our prenatal patients and making sure that we followed up on like their anatomy scan or their third trimester screening or like whatever shenanigans they need done. And then that would also flag if somebody has kind of been missing from care. But we don't have something like that in place for postpartum, but we probably should. *Participant 10, MD, Family Medicine*

Referral protocols were suggested specifically to overcome concerns related to credentialing. One participant explained:

There's a difference to me of being a screening place, a place of screening versus a place of management… I don't know that I would think that a pediatrician could manage hypertension in a newly postpartum patient, but I definitely think that there's processes that could be put into place to safeguard – to have that be the place of detecting it and having it be able to be escalated to the right place to manage it. *Participant 13, MD, Obstetrics*

The importance of opportunities to connect directly with other teams was highlighted in the following quote about co-location:

We have so many external resources that are truly wonderful, but our collaboration with them is not as good as when it's someone who's in our office… Learning each other's styles and learning how everyone works and operates. *Participant 20, MD, Pediatrics*

## Discussion

This article describes clinician perspectives on integrated interconception dyad care, including barriers to integration and applicability of existing structures and processes to improve dyad care. Clinicians noted multiple barriers to integrated dyad care and particularly emphasized the difficulty of communicating across health care settings. Consistent with prior work, participants noted that challenges with communication are also a barrier to integrated care for individuals.^25,26^

Dyad-specific barriers included the ways in which privacy, credentialing, and visit design focus on care for individuals. Importantly, when suggesting strategies to improve integration for dyads, participants referred to strategies already in use for health care improvement. This suggests that progress toward dyad integration may be relatively low cost, achievable with existing technologies and skills. Of note, although our interviews focused on care after preterm birth, participants did not suggest limiting dyad services to high-risk populations.

The substantial barriers described here may, in part, explain the slow uptake of integrated dyad care. Teen-Tot clinics, which provide health care services to young mothers and their infants in the same location with the same team, have been described for at least 30 years, and have demonstrated benefits, including decreased emergency department use and repeat teen pregnancies, and increased infant immunization and teen preventive care.^27–30^ Yet most young parents do not receive care through integrated models.^30^

Emerging programs have also considered dyad approaches for families affected by opioid use or after gestational diabetes.^31,32^ These programs emphasize some features in common with recommendations from our participants, including roles to support navigation and collocation of services.^31,32^ One program was developed with reference to the chronic care model. The chronic care model has been widely applied to health system redesign and is linked with care management.

This application of an existing model to dyad needs suggests, consistent with our findings, that available strategies may help improve dyad care integration.^14,33^ Establishing common features of dyad programs may support standardized approaches to implementing and assessing these models, ultimately building a more generalizable evidence base for integrated care.

Our findings suggest several strategies that health systems could readily apply to promote integrated dyad care. First, health systems could address barriers related to privacy by clarifying guidelines on records access and use when caring for mother–infant dyads. These guidelines would benefit from input by parents. We are unaware of any prior research on parent perspectives on this topic, although other findings from this research project suggest parental hesitation about full information sharing across maternal and infant teams. (Gregory EF, Beidas R, Fiks AG, et al. Acceptability of Dyad Care Management after Preterm Birth: A Qualitative Study. In review).

Second, health systems could work across divisions to develop referral practices to support screening in atypical settings (*e.g.,* maternal blood pressure screening in pediatrics). Third, health systems could invest in new roles specifically tasked with managing communication and accountability for care across settings. These roles would need appropriate access to records for both members of the dyad and supervision from both adult and pediatric teams.

Participants did not specify a credential for these roles, which could be created for care managers, navigators, or community health workers. Fourth, for clinical teams already caring for both mothers and infants (*i.e.,* Family Medicine settings, Teen-Tot Clinics), offices should consider redesigning visits to facilitate delivery of care to both members of the dyad. For example, previsit screening for maternal needs before frequent infant visits could allow scheduling extended visits to address maternal needs, if present. Payer policies and incentives around billing could accelerate adoption of these strategies.

This study had several limitations. First, our sample was limited to two academic health systems and may not reflect feasibility of dyad care at other systems or in community sites. In addition, we focused on a convenience sample of clinical team members who routinely provide care to birthing parents or infants. Other constituent groups in the health system, including patients and families, may have distinct perspectives. Of note, we did conduct corresponding interviews with birthing parents, generating minimal content on these themes.

Our current findings may inform future interviews in which parents are asked to reflect on the specific strategies suggested by clinicians. However, our sampling approach may have biased our findings. In some cases, we may overstate feasibility if our participants are predisposed to seek solutions for this population. Alternatively, we may understate feasibility as these individuals face daily challenges to integrating care. Despite potential biases, perspectives of experienced clinicians are critical to inform this emerging field.

Second, we appeared to have convergence; however, subgroup size was not designed to draw contrasts between departments or clinical roles. Third, we relied on a convenience sample and may not have represented all perspectives within the sampled groups. Finally, this qualitative study reflected participants reported beliefs about barriers and facilitators, but we cannot be sure that implementing the proposed strategies would improve dyad care.

## Conclusions

Although clinicians involved in maternal and infant care recognized unique barriers to integrating care for dyads, they also described one barrier, the challenge of communicating across health care settings, as analogous to challenges faced in care of individual patients. Participants suggested existing health care strategies can be applied to improve integration of interconception care for dyads. Despite substantial barriers, findings suggest feasible opportunities to improve delivery of interconception preventive services through dyad integration.

## Abbreviations Used

AHRQ: Agency for Healthcare Research and Quality
EHR: electronic health record
HIPAA: Health Insurance Portability and Accountably Act
LARC: Long Acting Reversible Contraception
MAs: Medical Assistant
NICU: neonatal intensive care unit
OB: obstetrician
RSV: Respiratory Syncytial Virus
